# Activity and Health During the SARS-CoV2 Pandemic (ASAP): Study Protocol for a Multi-National Network Trial

**DOI:** 10.3389/fmed.2020.00302

**Published:** 2020-06-05

**Authors:** Jan Wilke, Lisa Mohr, Adam S. Tenforde, Oliver Vogel, Luiz Hespanhol, Lutz Vogt, Evert Verhagen, Karsten Hollander

**Affiliations:** ^1^Department of Sports Medicine, Goethe University Frankfurt, Frankfurt, Germany; ^2^Department of Physical Medicine and Rehabilitation, Spaulding Rehabilitation Hospital, Harvard Medical School, Charlestown, MA, United States; ^3^Master's and Doctoral Programs in Physical Therapy, Universidade Cidade de São Paulo, São Paulo, Brazil; ^4^Amsterdam Collaboration on Health and Safety in Sports, Department of Public and Occupational Health, Amsterdam Movement Sciences, Amsterdam UMC, Amsterdam, Netherlands; ^5^University Medical Centers-Vrije Universiteit Amsterdam, Amsterdam, Netherlands; ^6^Medical School Hamburg, Hamburg, Germany

**Keywords:** physical activity, coronavirus, exercise, isolation, home-based, e-health

## Abstract

**Introduction:** The worldwide spread of the novel coronavirus (SARS-CoV2) has prompted numerous countries to restrict public life. Related measures, such as limits on social gatherings, business closures, or lockdowns, are expected to considerably reduce the individual opportunities to move outside the home. As physical activity (PA) and sport participation significantly contribute to health, this study has two objectives. The objectives of this study are to assess changes in PA and well-being since the coronavirus outbreak in affected countries. Additionally, we will evaluate the impact of digital home-based exercise programs on PA as well as physical and mental health outcomes.

**Method:** A multinational network trial will be conducted with three planned phases (A, B, and C). Part A consists of administering a structured survey. It investigates changes in PA levels and health during the coronavirus outbreak and measures the preferences of the participants regarding online training programs. Part B is a two-armed randomized-controlled trial. Participants assigned to the intervention group (IG) will complete a digital 4-week home exercise training (live streaming via internet) guided by the survey results on content and time of program. The control group (CG) will not receive the program. Part C is 4-week access of both CG and IG to a digital archive of pre-recorded workouts from Part B. Similar to Part A, questionnaires will be used in both Part B and C to estimate the effects of exercise on measures of mental and physical health.

**Results and Discussion:** The ASAP project will provide valuable insights into the importance of PA during a global pandemic. Our initial survey is the first to determine how governmental confinement measures impact bodily and mental well-being. Based on the results, the intervention studies will be unique to address health problems potentially arising from losses in PA. If proven effective, the newly developed telehealth programs could become a significant and easy-to-distribute factor in combating PA decreases. Results of the study may hence guide policy makers on methods to maintain PA and health when being forced to restrict public life.

**Study Register:** DRKS00021273.

## Introduction

Abundant evidence supports the value of physical activity (PA) and exercise as essential cornerstones of physical and mental health (1–3). For instance, it has been shown that regular movement lowers all-cause mortality by up to 80% while decreasing the odds of developing cardiovascular, neurological, musculoskeletal or psychiatric diseases (4). In view of these effects, specific guidelines detailing optimal PA have been developed for a variety of populations including children or older adults (5, 6) and health professionals and policy makers strive to implement them with considerable effort (7–10).

Since the outbreak of the novel coronavirus (SARS-CoV-2) in December 2019 and the classification as a global pandemic in March 2020, the opportunities to engage in sport and exercise have been greatly limited (11). Due to governmental regulations that restrict activities in public life [e.g., bans of public gatherings, business closures or city lockdowns; (12)], the ability to move freely has been reduced for the general population. Similar to initial actions in China, various countries (among others, United States of America, France, Germany, Spain, United Kingdom, and Italy) have taken measures that limit activities. The restrictions in access to sports clubs, gyms, and self-organized outdoor activities are assumed to result in a considerable decrease in global and individual PA levels (11).

Reductions in PA are not only relevant because of the unexploited benefits of regular movement. Inactivity and sedentary behavior, characterized as time spent in sitting, lying or reclined posture at low energy expenditures, have substantial adverse effects on health (13). A meta-analysis, pooling data from more than 1.3 million participants, demonstrated that particularly sitting and TV viewing time are both strongly associated with premature death (13). Such activities and other sedentary behavior may increase in populations affected by the coronavirus pandemic.

Government measures that aimed to control illness after the virus outbreak in China limited movement for millions of people over weeks to months (12). As other countries with registered cases implemented restrictive measures too, it is of the utmost importance to understand how such restrictions will change PA, physical health and mental well-being. Further, novel strategies may be required to maintain or improve PA at home. The objectives of our study are to examine the effects of public restrictions by geography on (a) PA and (b) individual well-being using an international population-based survey. Using these results, we plan to investigate the feasibility of digital home-exercise programs as well as their effectiveness in increasing physical and mental health.

## Materials and Methods

### Ethical Standard and Study Design

**Figure 1:**
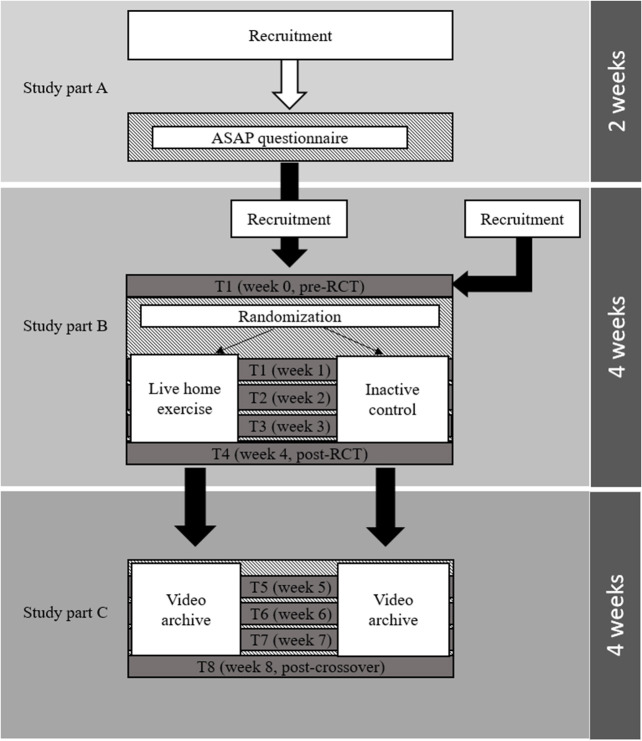
Visualization of the project flow in each center. In study part A, participants are recruited for a cross-sectional survey (ASAP questionnaire). Upon completion they are made aware about the opportunity of registering for the randomized, controlled trial (RCT, study part B). Participants are also recruited among individuals who have not completed the survey. After completing the RCT, participants will receive access to a video database for another 4 weeks (study part C).

The ASAP (**A**ctivity and health during the **SA**rs-CoV2 **P**andemic) project (Figure 1) consists of a structured, multinational cross-sectional survey (study Part A), a two-armed, randomized-controlled, multicenter parallel group trial (study Part B), and a controlled multicenter crossover trial (study Part C). It will be conducted according to the Guidelines of Good Clinical Practice and adhering to the Declaration of Helsinki. This study protocol reports according to the Standard Protocol Items: Recommendations for Interventional Trials (SPIRIT) guidelines (14). Approvals are obtained from the study center's review board (Ethics committee of the faculty of psychology and sport sciences at Goethe-University Frankfurt) as well as from all universities actively included into participant recruitment. The intervention parts of the study have been prospectively registered at the German Registry of Clinical Trials (DRKS00021273).

All participants will provide informed consent. Outcomes in all three portions of the study (Part A, B, and C) are assessed using digital questionnaires. After being provided with information on the investigation including purpose, aims, voluntary nature of participation and data use on the first page of the questionnaires, each individual will be asked to choose whether to select the “Participate” button, which signalizes digital consent to participate in the study. All data will be either collected anonymously without patient identifiers (survey for study Part A) or retrospectively anonymized (Parts B and C).

### Participants

The target population will include residents aged 18 and older from countries with (1) officially registered cases of the novel coronavirus (SARS-CoV-2) and (2) active governmental restrictions limiting public life via bans of public gatherings, forced restrictions of social, contact business closures, or lockouts. Recruitment will be performed by means of advertising in social media platforms (e.g., Youtube, Facebook, Twitter, Instagram) as well as health-related institutions (e.g., national chapters of the Exercise is Medicine initiative).

### Procedures and Interventions

#### Study Part A

In the first part of the project, a structured multi-national survey will be administered during a 2-week period. The ASAP questionnaire is answered digitally and requires about 5–10 min to complete.

The survey instrument has four sections. The first portion assesses demographic data including age, sex and country of residence. The second section captures self-reported physical activity levels and exercise habits prior to and since the outbreak of the coronavirus. The questions have been newly constructed or adopted from valid measures in order to account for the specificities of the situation. Physical activity levels will be assessed using the Nordic Physical Activity Questionnaire-short (NPAQ-short, 15). The 2-item instrument measures the total time spent in free time during moderate to vigorous physical activities and during vigorous physical activities only. The questions were adapted to also account for working/occupational time. The NPAQ-SF has been shown to be reliable and was validated to monitor compliance with the WHO recommendations on physical activity (15). The third section of the ASAP questionnaire addresses the physical and mental well-being of the participants, again comparing the situation before and after the outbreak. Also, this part consists of questions newly constructed as well as psychometrically validated and cross-culturally adapted questionnaires. Regarding the latter, bodily pain is assessed using the sub-scale of the SF-36 questionnaire and mental well-being is measured using the WHO5-scale (16, 17). In the final section, we examine the preferences of the participants for exercise programs that will be developed based on the answers (e.g., total time, type(s) of exercise and activity).

The ASAP questionnaire was developed using an expert consensus process similar to that described in a previous investigation (18). Briefly, after agreeing on the scope and contents of the questionnaire, an initial version of the instrument was independently reviewed by the consensus team members which included physicians, physiotherapists, movement scientists, and sports scientists. Their blinded feedback was used to refine the questionnaire. For content validation, the questionnaire was sent to experts from different professions not belonging to the research team involved in its development (19). To increase face validity, members of the target population without background from a health profession were asked to provide feedback on comprehensibility and clarity of the questionnaire (20). The assessment tool is available in seven languages [Dutch, English, German, French, Italian, (Brazilian) Portuguese, Spanish]. Clarity and comprehensibility have been validated via forward and back translation by native speakers.

#### Study Part B

Based on the results of study part A, the second part will consist of a multicenter, two-armed, randomized-controlled parallel group trial. Participants in the intervention group (IG), for a period of 4 weeks, will receive online workouts with video live-streaming using the appropriate software (e.g., Zoom, Zoom video communications, San Jose, California, USA; BlackBoard, Washington, DC, USA). Duration, frequency, and contents will be selected balancing (a) the needs of the population as indicated via the ASAP questionnaire and (b) scientific recommendations for exercise prescription. For example, the minimum training frequency will be once per week and minimum duration will be 10 min (21). To allow a higher degree of standardization between the countries, the instructors will be provided with modifiable demo workouts exhibiting different content-related focuses (e.g., strength, endurance, postural control/balance, cognition, relaxation), which can be individually adapted. The control group (CG) will not receive an intervention and is instructed to complete the outcome assessments (see below). Randomization (1:1 ratio) will be performed using a software algorithm of the online database used for survey delivery (Soscisurvey, Soscisurvey GmbH, Munich, Germany). To allow concealed group allocation, the participants will be automatically informed by the system about allocation upon survey completion at baseline.

A two-fold approach is used for recruitment. Firstly, upon completion of the ASAP questionnaire (study part A), each participant will be informed about the opportunity to participate in the subsequent intervention trials (Study Parts B and C). Second, the same recruitment strategies used for the initial survey (social media advertising and promotion via associations and societies) will be used to enhance recruitment.

#### Study Part C

Study Part C adopts a controlled crossover design. Following completion of the post-measurements of study Part B, the participants of both groups (intervention and control) will receive access to an online database of recorded workouts with contents similar to Part B. All contents can be freely used for four additional weeks.

### Outcomes

As indicated above, the ASAP questionnaire represents the outcome of interest for study Part A. For study Parts B and C, eight assessments are planned: at baseline prior to the RCT (T1), as well as weekly during the RCT (T2–T5) and the crossover study (T6–T8). Each survey will include an assessment of basic information (e.g., sex and age) and brief questions assessing general psychological and physical well-being. Additionally, a battery of questionnaires will be applied. The components were chosen based on both, thorough psychometric evaluation and the availability of translation and cross-cultural validation for the languages used. Implemented tools include the WHO5 scale for mental well-being (16, 17), generalized anxiety disorder scale-7 [GAD-7, (22)] for impulsiveness and anxiety, the MOS 12-item scale for sleep quality (23), the self-concordance scale (24) for exercise motivation and the Chronic Graded Pain Scale (25) for pain. In addition to the intervention effects, data on acceptance and adherence will be collected by means of documenting attendance at each workout offered in study Part B as well as by means of asking for the frequency of database use in study Part C (T4 assessment).

### Data Processing and Statistics

All datasets will be analyzed using intention-to-treat. The findings from the ASAP questionnaire (Study Part A) will be descriptively reported and presented using appropriate measures such as mean ± standard deviation or median and interquartile range depending on distributions and scales of measurement. Additionally, the significance of variable associations (e.g., between physical activity levels and markers of well-being) will be examined using correlation and regression analyses.

To estimate the risk of non-response bias, wave analyses will be conducted according to Lewis et al. (26). Specifically, the responses of the first 10% percent of the participants (early responders) will be compared to those of the last 10% (late responders) by means of inferential statistics. The rationale behind this is that early responders are assumed to be more motivated than late responders which can be compared to non-responders. Hence, if the wave analyses do not provide significant findings, absence of non-response bias is concluded.

For study Parts B and C (randomized, controlled trial/controlled crossover trial), a prospective meta-experiment approach will be applied (27). For each country, the mean pre-post-differences between-groups including 95% confidence intervals (95% CI) will be calculated at the different time points. An a priori sample size calculation using an algorithm specifically designed to account for between-site variance in multi-center trials was performed (28). When achieving a sample size of *n* = 544 with an included drop-out rate of 20%, the trial will have 80.3% power to detect pre-post-differences with a minimal effect size (Cohen's *d*) of 0.25 at an alpha level of 0.05. To account for potential between-center variance, the data collected in each country will be pooled using a random-effects model (29). This leads to an aggregated effect size (weighted mean differences) demonstrating the overall effectiveness of the intervention while the different countries can still be compared by means of inspecting the 95% CI's. Heterogeneity between countries will be quantified by means of the *I*^2^ index (30). To further explore its potential sources (e.g., country, age, sex, baseline physical activity), a meta-regression with continuous and factorial independent variables will be performed (31).

Data analyses will be performed using standard statistical software packages (e.g., SPSS 22, SPSS Inc., Chicago, Illinois, USA and BiAs statistics, Goethe University, Frankfurt/Main, Germany). The significance level for all analyses will be set to α = 0.05.

## Discussion

Restricting the opportunities to move outside the own home, while important to control the spread of the novel coronavirus, may limit PA. Our study aims to understand the influence of forced social isolation during the pandemic on movement habits and markers of self-reported mental and physical health.

To date, most research on the novel coronavirus has focused on the crucial topics of detection and treatment, including diagnostic measures, vaccines, and therapeutic pharmaceuticals (32–34). However, it may be argued that the adverse effects of the pandemic extend beyond the direct consequences of infection with SARS-Cov2. Since millennia, the engagement in physical activity and exercise represent significant contributors to human health and compelling evidence has demonstrated its benefits (1–3, 35). As the protective and therapeutic effects, in many cases are similar or superior to pharmaceutic remedies, some have considered exercise to represent a drug which is free of charge while exhibiting a favorable side effect profile (4, 36, 37). The outbreak of the novel coronavirus has both threatened the availability of medical devices and pharmaceutical remedies (38, 39), but also that of exercise medicine: restricting the opportunities to move outside limits the feasibility and availability of physical activity and exercise. Our study, particularly part A (ASAP survey), therefore, will provide relevant data gauging the influence of forced social isolation during the pandemic.

Based on the findings of the cross-sectional questionnaire assessment, the prospective study Parts 2 and 3 will measure the effectiveness of home-based digital exercise programs in addressing limitations in PA and well-being during the pandemic. In first line, they may help counteract the negative bodily effects of inactivity (e.g., musculoskeletal pain, increased risk of cardiovascular diseases, weight gain). In addition, while speculative, participation could also have an indirect effect on the pandemic. An analysis of previous influenza virus infections demonstrated that individuals who rarely or never work out have a reported 6 to 9 percent higher mortality risk (40). This is consistent with studies showing that acute bouts of moderate exercise (65–70% of VO_2_ peak) increase the levels of cytokines (i.e., Interleukin-6) needed during immune response (4, 41, 42). In sum, this could suggest that exercise has a protective effect against viral infections although further research is needed to understand the role of exercise in modifying disease from the novel coronavirus.

The planned interventions may also be of relevance from psychological and political perspectives. Social isolation has been demonstrated to have a detrimental influence on a variety of mental health markers. For instance, loneliness leads to mood changes, depression and increased overall mortality (43, 44). Initial evidence for the COVID-19 pandemic shows that life satisfaction decreased in Chinese adults forced to stop working (45). As exercise has positive effects on psychological well-being (3, 35, 46), it may help improve the capacity to deal with the current situation. From a theoretical point of view, the success of governmental restrictions in public life will depend on both their execution and control but also on the compliance of the population. Improving coping by means of sport may thus help governmental goals to maintain restrictions and to control contagion.

Some methodological considerations are needed. As home-exercise may become an important method to maintain PA during future confinements, it will be particularly interesting to study adherence. It has been reported that the feeling of being supported and the possibility to contacting the provider may facilitate compliance (47). As our exercises in study part A will be live-streamed and the participants can interact with the instructors, we believe this can improve training frequency compared to traditional home-exercise programs. Compliance will also be of importance in our CG. As it does not receive an intervention, participants may withdraw from the study. We chose two strategies to counteract this. Firstly, we offer them free database use in study Part B and thus, any participant enrolled will have a PA intervention. Secondly, the CG participants will be actively motivated to express their preferences regarding the video-database and, using their feedback, some workouts will be specifically tailored for them. Besides compliance, another issue relates to outcome assessment. We decided to use questionnaire assessments in both study parts, which is congruent with the objective to measure and improve subjective well-being and allows the achievement of large sample sizes. However, regarding PA assessments, it should also be noted that most persons tend to overestimate the own activity levels and that the recall of moderate-intensity activities is less precise than that of vigorous activities (48).

## Author Contributions

JW, LM, KH, LV, LH, EV, OV, and AT: conception and design. JW and KH: drafting of the manuscript. JW, LM, KH, LV, LH, EV, OV, and AT: critical revision and proofreading. All authors: contributed to the article and approved the submitted version.

## Conflict of Interest

The authors declare that the research was conducted in the absence of any commercial or financial relationships that could be construed as a potential conflict of interest.
